# The UBE2D ubiquitin conjugating enzymes: Potential regulatory hubs in development, disease and evolution

**DOI:** 10.3389/fcell.2022.1058751

**Published:** 2022-12-12

**Authors:** Monica Roman-Trufero, Niall Dillon

**Affiliations:** ^1^ Centre for Haematology, Department of Immunology and Inflammation, Imperial College, Hammersmith Hospital Campus, London, United Kingdom; ^2^ MRC London Institute of Medical Sciences, Imperial College, Hammersmith Hospital Campus, London, United Kingdom

**Keywords:** ubiquitination, UBE2D3, phosphorylation, development, evolution

## Abstract

Ubiquitination of cellular proteins plays critical roles in key signalling pathways and in the regulation of protein turnover in eukaryotic cells. E2 ubiquitin conjugating enzymes function as essential intermediates in ubiquitination reactions by acting as ubiquitin donors for the E3 ubiquitin ligase enzymes that confer substrate specificity. The members of the UBE2D family of E2 enzymes are involved in regulating signalling cascades through ubiquitination of target proteins that include receptor tyrosine kinases (RTKs) and components of the Hedgehog, TGFβ and NFκB pathways. UBE2D enzymes also function in transcriptional control by acting as donors for ubiquitination of histone tails by the Polycomb protein Ring1B and the DNA methylation regulator UHRF1 as well as having roles in DNA repair and regulation of the level of the tumour suppressor p53. Here we review the functional roles and mechanisms of regulation of the UBE2D proteins including recent evidence that regulation of the level of UBE2D3 is critical for controlling ubiquitination of specific targets during development. Cellular levels of UBE2D3 have been shown to be regulated by phosphorylation, which affects folding of the protein, reducing its stability. Specific variations in the otherwise highly conserved UBE2D3 protein sequence in amniotes and in a subgroup of teleost fishes, the Acanthomorpha, suggest that the enzyme has had important roles during vertebrate evolution.

## Introduction

Ubiquitination is an ancient post-translational modification that is found in all eukaryotic lineages (Grau-Bove et al., 2015). It occurs through covalent attachment of a small 8.6 kd protein (ubiquitin) at specific residues on target proteins. The attachment sites can be lysine, cysteine, methionine, serine, threonine or N-terminal residues of target proteins (reviewed by Swatek and Komander, 2016). Ubiquitin residues can be attached as single molecules (monoubiquitination), or as linear or branched chains (polyubiquitination). The use of different ubiquitin residues for branch formation allows complex branching of polyubiquitin chains, increasing the potential versatility of this modification as a recognition signal (Komander and Rape, 2012). Monoubiquitination has major signalling roles in the cell (Magits and Sablina, 2022), whereas the primary function of polyubiquitination is to target proteins for destruction by the proteasome (Grice and Nathan, 2016). The multiple regulatory functions of ubiquitination and, particularly, its role in controlling degradation of almost all cellular proteins, give it a unique significance in eukaryotic cell biology. This review will focus on the UBE2D family of E2 ubiquitin conjugating enzymes, which act as key intermediates in a wide range of ubiquitination reactions that impact on development, cellular homeostasis and disease. We will also review emerging evidence that post-translational modifications have an important role in regulating UBE2D enzyme functions.

## E2 enzymes are key intermediates in ubiquitination with critical roles in health and disease

**FIGURE 1:**
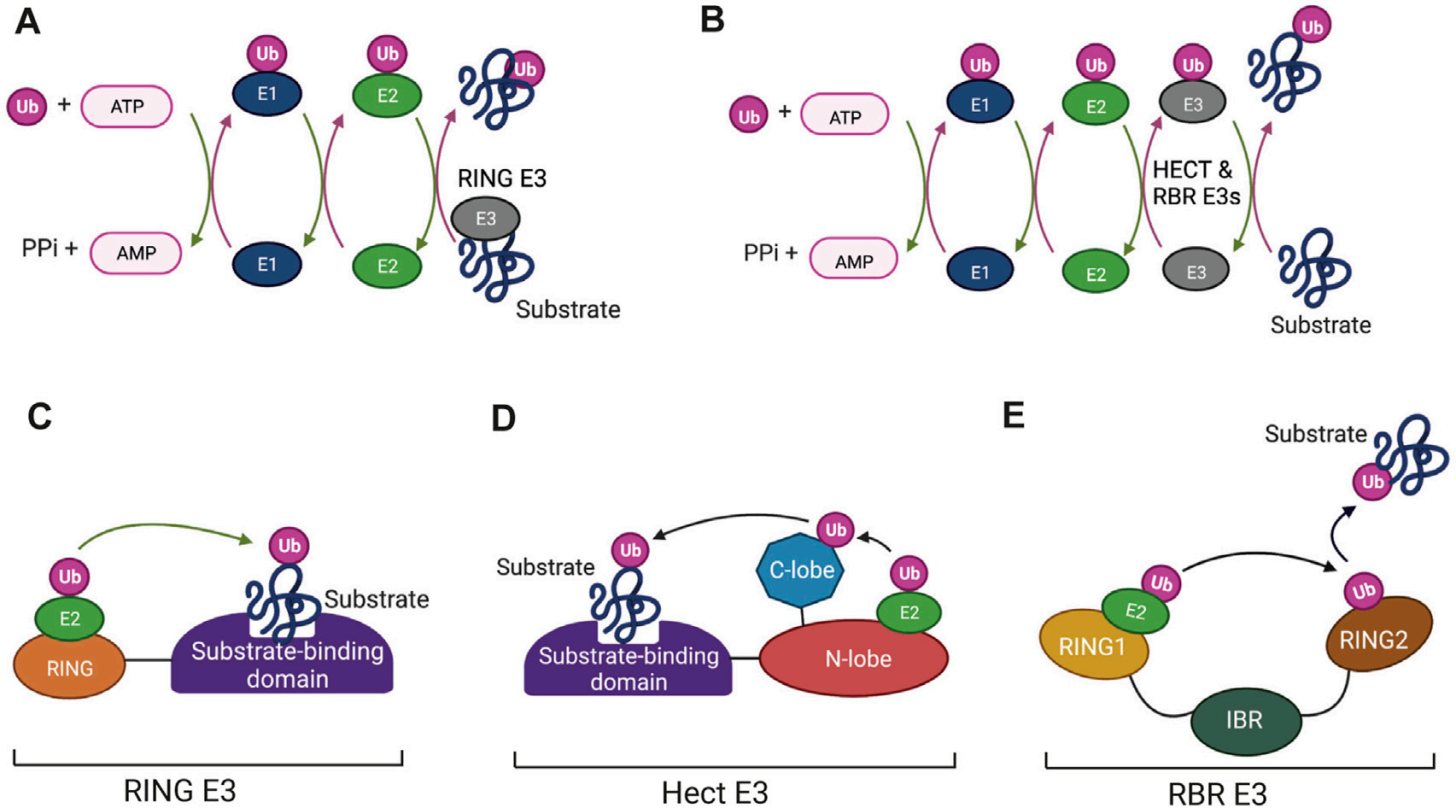
The enzymes that mediate protein ubiquitination: **(A,B)** The enzyme cycles for ubiquitination involving RING finger E3 ligases (A) and HECT and RING-Between-RING (RBR) E3s (B). Ubiquitin is first activated by the ATP-dependent formation of a thioester bond to a cysteine residue in the E1 enzyme. This is followed by conjugation of the activated ubiquitin to the active-site cysteine of the E2 enzyme *via* a transthiolation reaction. Binding of E2-Ub conjugates to RING finger E3s (A) allows direct transfer of Ub to bound substrates. Ubiquitination mediated by HECT and RBR E3s (B) involves formation of transient thioester intermediates between ubiquitin and cysteine residues in the E3s followed by transfer from the E3 to the protein substrates. **(C–E)** Domain structures of the different categories of E3. **(C)** RING finger E3s consist of a RING domain that binds the E2-UB conjugate and positions it for transfer to the substrate. **(D)** HECT E3s contain a HECT domain, which consists of N-terminal and C-terminal lobes. The E2-Ub conjugate binds to the N-lobe and the ubiquitin then forms a thioester bond with a conserved catalytic cysteine in the C-lobe followed by transfer to the bound substrate. **(E)** RBR E3s consist of a canonical RING domain (RING1), an In-Between-RING (IBR) domain and a non-canonical RING domain (RING2). The E2-Ub conjugate binds to RING1 and the flexible inter-domain regions facilitate formation of the intermediate thioester linkage between Ub and a catalytic cysteine located in the RING2 domain. Ub is then transferred from the RING2 domain to the substrate by as yet undetermined mechanisms. Figure created with BioRender.com.

The enzymatic process that results in attachment of ubiquitin to its target molecule is a pyramid in terms of the numbers of different enzymes that are involved in the process (shown schematically in Figure 1). An E1 enzyme activates ubiquitin by forming an E1-ubiquitin thioester bond. The activated ubiquitin is then transferred to a cysteine residue at the active site an E2 enzyme, which in turn acts as a donor for transfer of the ubiquitin to the target protein in a reaction that is catalysed by the E3 ligase enzymes (Figures 1A, B). RING-type E3 enzymes catalyse direct transfer from the E2 to the substrate (Figure 1C) whereas the HECT (Homologous to the E6-AP Carboxyl Terminus) domain E3s and the RBR (Ring-Between-RING) E3s, form an intermediate covalent bond between the ubiquitin molecule and the E3 enzyme (Figures 1D, E) (Deshaies and Joazeiro, 2009; Walden and Rittinger, 2018; Weber et al., 2019).

The pyramid-shaped organization of ubiquitination enzymes arises from the fact that eukaryotic cells typically have one or two E1 enzymes, an intermediate number of E2 enzymes (around 30 in vertebrates) and a much larger number of E3 enzymes (>1,000 in vertebrates). RING and HECT E3 enzymes typically have substrate binding domains (Figures 1C, D), which allow them to direct the E2 enzymes to specific target proteins. It has long been known that individual E2 enzymes are specific for different E3 reactions with each E2 potentially interacting with a suite of E3 “clients”. A consequence of this is that changes to the functioning of a single E2 can affect a number of different pathways by altering the activity of more than one E3 enzyme. Such changes have the potential to alter the cellular landscape with important effects on health and disease. Post-translational modifications in response to signalling pathways are one type of mechanism that could have this effect. E2 enzymes have been shown to be subject to modifications that include phosphorylation, acetylation, S-nitrosylation and self-ubiquitination (see below), but information about the effects of these modifications is still relatively limited. That fact that E2s are druggable (Ceccarelli et al., 2011; Liu et al., 2014) also makes them potentially interesting targets in human disease.

Some E3 enzymes have also been shown to use more than one E2 enzyme for ubiquitnation reactions. Examples of E3s that fall into this category include CBL which is involved in the regulation of growth factor receptors (Liyasova et al., 2019) (see below), BRCA1, which has key roles in DNA repair (Christensen et al., 2007), and the Anaphase-Promoting Complex (APC), which plays essential roles in regulating mitosis by triggering the transition from metaphase to anaphase (Rodrigo-Brenni and Morgan, 2007; Wu et al., 2010). Interactions with different E2s show partial redundancy raising the possibility that E2 enzymes with different affinities can compete for access to a single E3. In addition, the observation that different E2s can have preferences for either monoubiquitination, or the formation of specific types of branched polyubiquitin chains adds another layer of complexity to the regulation of ubiquitination (reviewed by Ye and Rape, 2009).

The APC/C E3 ligase complex provides a striking example of the complexity of interactions of an E3 with multiple E2 enzymes (reviewed by Watson et al., 2019). In human cells, APC/C uses three E2 enzymes as ubiquitin donors, UBE2C, UBE2S and UBE2D (Wu et al., 2010; Wild et al., 2016). UBE2D is the donor for the inital monoubiquitination of substrates and UBE2C and UBE2S are used for subsequent K11-linked chain extension. Depletion of UBE2C and UBE2S reduces APC/C activity, but UBE2D mediated ubiquitination by the APC/C continues to occur at a lower level. This allows mitotic progression to continue, but switch-like metaphase to anaphase transition and dependence on the spindle assembly checkpoint are no longer observed (Wild et al., 2016).

## Structure-function relationships of the UBE2D enzymes

**FIGURE 2:**
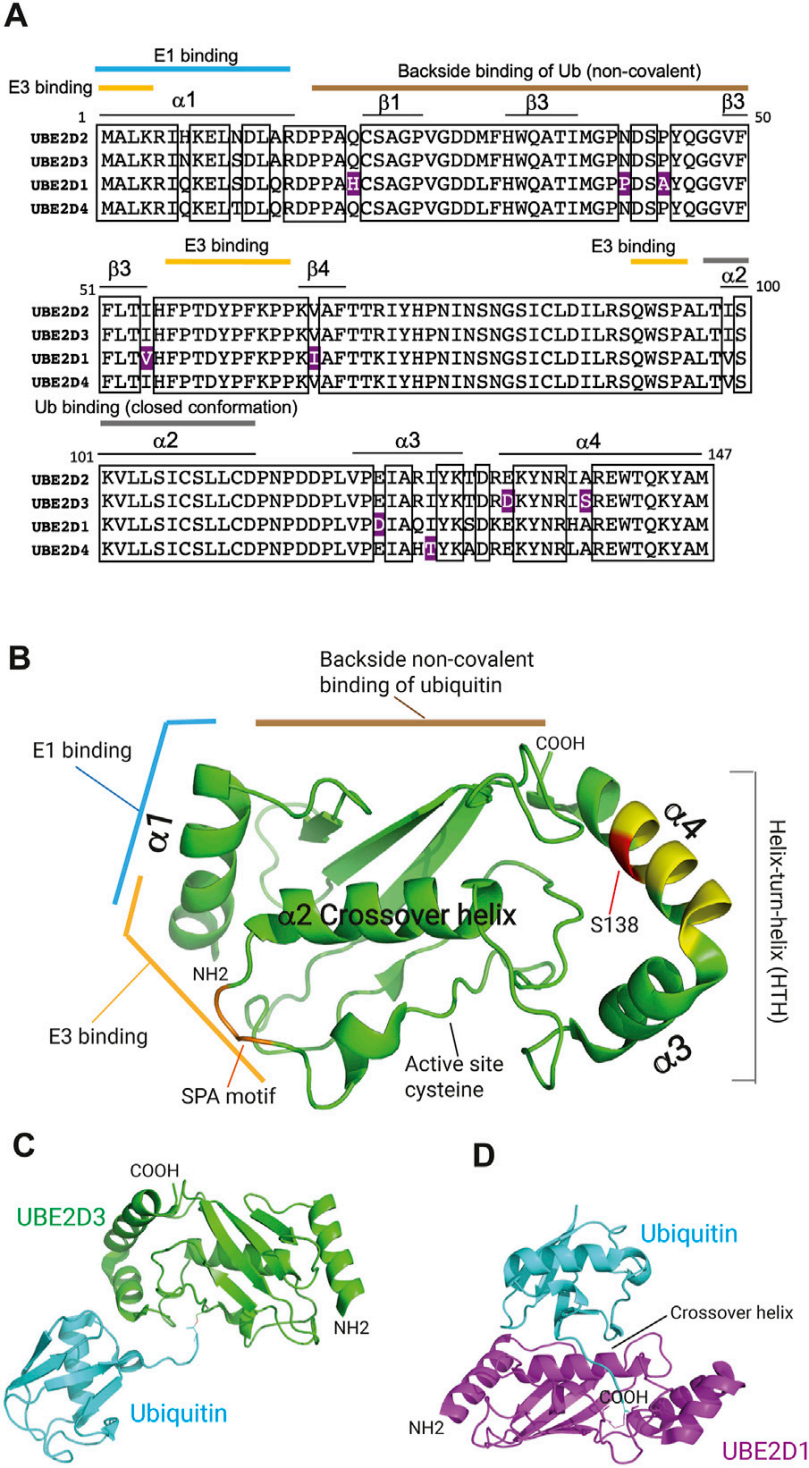
Domain structure of the UBE2D ubiquitin conjugating enzymes. **(A)** Comparison of the protein sequences of the UBE2D enzymes in humans. Boxes indicate the sequences that are identical in all four enzymes. Residues that are divergent in only one of the UBE2D proteins and conserved in the other three are highlighted in purple. UBE2D2 and UBE2D3 show the greatest similarity, diverging from one another in only three residues (98% identity), whereas UBE2D1 shows the largest divergence from the sequences of the other three enzymes (4%). The positions of α-helices and β-sheet regions are also shown, as are the regions that mediate binding of the E1 and E3 enzymes, the non-covalent backside binding of ubiquitin (Ub), and the interaction with the α2 crossover helix, which generates the closed active conformation of ubiquitin. **(B)** Location of binding domains in the 3-dimensional structure of UBE2D3 (Stewart et al., 2016). The positions of the residues that are hypervariable in UBE2D3 in Percomorph fish species (Figure 4) are indicated in yellow and the position of the amniote-specific UBE2D3-Ser138 residue is shown in red. The SPA motif is coloured orange. Image was prepared by visualising PDB: 2FUH using PyMol (v2.5.4). **(C)** Structure of the UBE2D3-ubiquitin conjugate with ubiquitin in the extended configuration (PDB 3UGB). UBE2D3 and ubiquitin are coloured green and cyan respectively. **(D)** Structure of the UBE2D1-ubiquitin conjugate bound to the RNF4 E3 ligase (not shown) with ubiquitin in the closed “active” configuration generated by interaction with the α2 crossover helix of UBE2D1 (PDB: 4AP4). UBE2D1 is shown in magenta and ubiquitin in cyan.

All E2 ubiquitin conjugating enzymes have in common a central catalytic domain, the UBC domain, which contains the active site cysteine. In addition to this universal domain, some E2s have amino terminal extensions, C-terminal extensions, insertions, or combinations of any of these three features (reviewed by Stewart et al., 2016). The UBE2D family of enzymes are canonical examples of vertebrate UBC-only E2 enzymes and have been used to investigate many of the properties of this class of E2. There are three UBE2D family members, UBE2D1, UBE2D2 and UBE2D3 (also known as Ubch5a, Ubch5b and Ubch5c) that are broadly distributed across vertebrate lineages and a fourth, UBE2D4 (Ubch5d), which has a more restricted distribution. The four UBE2D enzymes show very high (95%–98%) homology with one another (see Figure 2A and accompanying legend). Structural analysis has shown that each contains four α-helical and four β-sheet regions (Figures 2A, B). Domains of binding of E1 and E3 enzymes have been identified, along with non-covalent interaction of free ubiquitin with the “backside” region of the E2 (Stewart et al., 2016) (Figures 2A, B). Backside binding of ubiquitin to UBE2D enzymes has been shown to facilitate self-assembly of UBE2D-ubiquitin conjugates into larger complexes, which in turn promotes processive assembly of polyubiquitin chains (Brzovic et al., 2006; Sakata et al., 2010; Page et al., 2012).

Evidence from structural and functional studies indicates that free UBE2D-ubiquitin conjugate adopts an extended “inactive” configuration, whereas binding of an E3 enzyme results in a change to a closed “active” configuration, which places the active site cysteine in a favourable position for transfer to the substrate (Figures 2C, D) (Pruneda et al., 2011; Dou et al., 2012; Plechanovova et al., 2012). Recent work has indicated that interactions of conjugated ubiquitin with the helix-turn-helix (HTH) region of UBC-only E3s can promote formation of the open inactive state over the closed state (Welsh et al., 2022). Modulation of this interaction through sequence variation provides an additional potential mechanism for regulating the activity of different E2s (Welsh et al., 2022).

**FIGURE 3:**
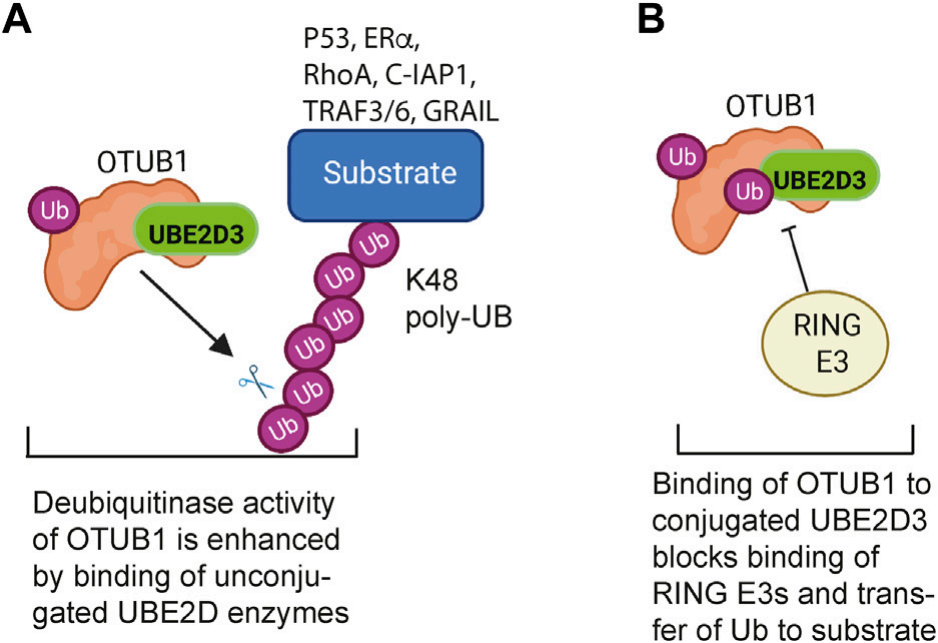
Regulatory interactions between the UBE2D enzymes and the deubiquitinase OTUB1. **(A)** Canonical regulation of ubiquitination by OTUB1: The deubiquitinase activity of OTUB1 is specific for K48-linked polyubiquitin chains and is potentiated by binding of a number of unconjugated E2 enzymes, including UBE2D3. OTUB1 antagonises K48-linked polyubiquitination and degradation of substrate proteins that regulate cell proliferation, apoptosis, TNF signalling and T cell immune responses. **(B)** Non-canonical inhibition of ubiquitination by OTUB1: Binding of ubiquitin-conjugated UBE2D3 to OTUB1 blocks binding of RING E3 ligases to UBE2D3, preventing the E2 from functioning as a ubiquitin donor. OTUB1 has a similar inhibitory effect on UBE2N (UBC13). Binding of unconjugated ubiquitin to OTUB1 is potentiated by binding of free ubiquitin to a second, distal, site. Figure created with BioRender.com.

Interaction with regulatory proteins is another mechanism for modulating the activity of the UBE2D enzymes. The best characterised example of this is the interaction with OTU-containing ubiquitin aldehyde-binding protein 1 (OTUB1), a deubiquitinase that is required for normal development and functioning of the lung in mice (Ruiz-Serrano et al., 2021). The canonical regulatory functions of OTUB1 reside in its deubiquitinase activity, which is specific for K48-linked polyubiquitin chains. The deubiquitinase activity is enhanced by binding of unconjugated UBE2D and UBE2N, to OTUB1 (Figure 3A) (Wiener et al., 2013; Que et al., 2020). In addition to this activity, OTUB1 also has a non-canonical regulatory activity, which involves sequestration of conjugated UBE2D, UNE2N and UBE2E enzymes in a configuration that blocks access of RING E3s to the E2-Ub conjugate, thereby preventing the E2 from acting as a ubiquitin donor (Figure 3B) (Nakada et al., 2010; Que et al., 2020). The A20/TNFAIP3 protein, which is a negative regulator of inflammation, has also been reported to bind to conjugated UBE2D3, promoting its degradation (Shembade et al., 2010), but the nature of the interaction has not been determined.

## The UBE2D proteins have multiple roles in vertebrate signalling pathways

**FIGURE 4:**
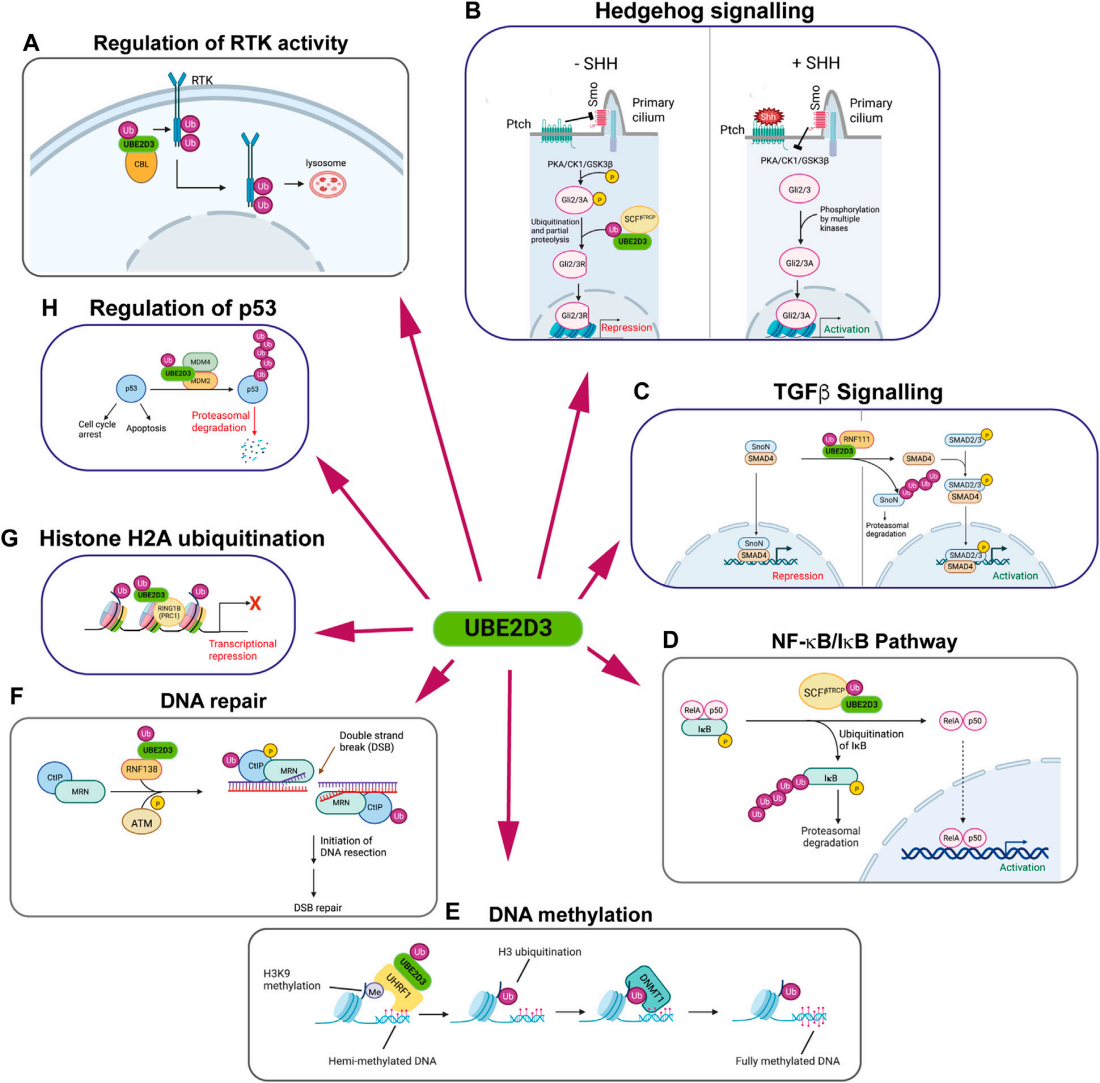
The multiple regulatory roles of the UBE2D enzymes. Panels show schematic representations of pathways that are regulated by UBE2D-mediated ubiquitination. UBE2D3 is shown, as it is the most highly expressed of the UBE2D enzymes in mammalian cells. Clockwise from top left: **(A)** Regulation of receptor tyrosine kinase (RTK) activity: Monoubiquitination of RTKs by the E3 ligase CBL using UBE2D3 as a ubiquitin donor results in internalisation and trafficking to the lysosome, where the RTKs are degraded. **(B)** Hedgehog signalling: In vertebrates, absence of Sonic Hedgehog (SHH) allows the patch receptor (Ptch) to inhibit the Smo receptor, which is located in the primary cilium. Blocking Smo allows PKA, CK1 and GSK3β to phosphorylate specific residues on the Gli2/Gli3 transcription factors. Phosphorylation promotes ubiquitination of Gli2/3 by the E3 ligase SCF^βTRCP^ in conjunction with UBE2D3, leading to partial proteolysis of Gli2/3. This converts Gli2/3 to repressors (Gli2/3R). Increased levels of SHH act on Ptch, blocking its effect on Smo and allowing it to inhibit PKA/CK1/GSK3β. This opens Gli2/3 to phosphorylation by CK2, Akt and other kinases, converting them to their active forms (Gli2/3A) and leading to activation of SHH target genes. **(C)** TGFβ signalling: Left: The repressor SnoN interacts with SMAD4 at target genes repressing their expression in the inner cell mass and developing epiblast cells of the mouse embryo. During primitive streak formation, the Ring finger E3 ligase RNF111 (Arkadia) interacts with the UBE2D enzymes to ubiquitinate SnoN1, targeting it for degradation and allowing activation of SMAD4 target genes. **(D)** NF-κB/IκB pathway: IκB forms an inhibitory complex with the NF-κB subunits RelA and p50. Phosphorylation of IκB promotes its ubiquitination by the E3 ligase RNF138 in conjunction with UBE2D3, leading to proteasomal degradation of IkB and activation of NF-κB target genes. **(E)** DNA methylation: Maintenance methylation of hemi-methylated DNA is promoted by binding of the E3 ligase UHRF1 to di- and tri-methylated H3K9. UHRF1 acts in conjunction with UBE2D3 to monoubiquitinate H3 at K14, K18, K23 and K27. The ubiquitinated H3 residues bind DNMT1, leading to methylation of the unmethylated DNA strand. **(F)** DNA repair: The ring finger E3 ligase RNF138 ubiquitinates the N-terminal region of the DNA repair protein CTiP using the UBE2D proteins as ubiquitin donors. N-terminal ubiquitination of CTiP facilitates the biding of the CTiP/MRN DNA repair complex to double strand breaks (DSBs) where it initiates DNA resection in the first step that leads to DSB repair. **(G)** Histone H2A ubiquitination: The Polycomb repressor complex-1 (PRC1) component Ring1B is an E3 ligase that uses UBE2D3 as a ubiquitin donor for monoubiquitination H2AK119. Ubiquitinated H2AK119 represses transcription of Polycomb target genes. **(H)** Regulation of p53: Polyubiquitination of p53 by the MDM2/MDM4 complex, in conjunction with UBE2D3, triggers proteasomal degradation. This results in modulation of the multiple activities of p53, including promotion of cell cycle arrest and apoptosis. Figure created with BioRender.com.

UBE2D enzymes are relatively promiscuous and act as ubiquitin donors for a wide range of E3 enzymes. Their known involvement in a number of key pathways that affect development and protein turnover makes them potentially significant contributors to cellular regulation (summarized in Figure 4). Some of these pathways will now be considered in more detail:

### Regulation of receptor protein tyrosine kinases (RTKs)

RTKs are involved in essential functions across all of the metazoan orders. They include platelet-derived growth factor receptor (PDFGR), epidermal growth factor receptor (EGFR) and the fibroblast growth factor receptors (FGFRs), all of which play critical roles in mammalian development and in human cancers. The casitas B-lineage lymphoma (CBL) proto-oncogene is an E3 ligase that has an important role in regulating RTK levels by ubiquitinating and downregulating a number of RTKs (Thien and Langdon, 2001; Mohapatra et al., 2013). CBL mediates monoubiquitination and polyubiquitination of activated EGFR, PDGFR and FGFRs (Figure 4). The E2 enzymes that have been shown to act as ubiquitin donors for these ubiquitination reactions include the UBE2D enzymes, which can act as donors for monoubiquitination and polyubiquitination by CBL, and UBE2N and UBE2W, which promote polyubiquitination and monoubiquitination respectively (Liyasova et al., 2019).

Monoubiquitination and polyubiquitination of RTKs have the effect of promoting trafficking of the RTKs to the lysososme where they are degraded, resulting in reduction in RTK levels at the cell surface and attenuation of signaling (Mohapatra et al., 2013) (Figure 4A). Knockdown of UBE2D3 and UBE2D4 using siRNAs was shown to give the largest reductions in EGF-induced EGFR ubiquitination in HeLa cells, confirming the role of these enzymes in *in vivo* regulation of RTK activity (Liyasova et al., 2019). Interestingly, the observation that knockdown of different E2s affects CBL functioning indicates that the E2s act non-redundantly in ways that are not fully understood.

### Hedgehog signalling

The Hedgehog morphogen plays a critical role in regulating a number of aspects of early development*. Drosophila* possesses a single Hedgehog isoform, which regulates formation of the larval imaginal wing discs, whereas three Hedgehog homologues have been identified in vertebrates. The most broadly expressed vertebrate isoform is Sonic Hedgehog (Shh), which is known to regulate a very wide range of processes during mammalian development. These include left-right axis formation, dorsoventral neural patterning, cranial neural crest patterning, limb, tooth and eye development and formation of the placenta (reviewed by (Briscoe and Therond, 2013).

Ubiquitination by the E3 ligase SCF^βTRCP^/Slimb using UBE2D family members (*Drosophila* ortholog UBCD1) as ubiquitin donors plays a central role in the regulatory mechanisms of Hedgehog signalling in flies and vertebrates. SCF^βTRCP^/Slimb-mediated ubiquitination triggers proteolytic degradation of the C-terminal regions of the transcription factor Cubitus interruptus (Ci) in flies and the Ci orthologues Gli2 and Gli3 in vertebrates, converting them from transcriptional activators to repressors (Ingham, 1998; Maniatis, 1999; Bocca et al., 2001; Briscoe and Therond, 2013; Pan et al., 2017). Ubiquitination of Ci/Gli proteins in *Drosophila* and in vertebrates is dependent on them being phosphorylated by the PKA, CK1 and GSK3β kinases, High levels of Hedgehog block this phosphorylation, leaving the proteins intact and allowing them to upregulate a cohort of Hedgehog target genes (Figure 4B). Hedgehog forms a morphogen gradient in the imaginal wing discs in *Drosophila* larvae and mutations in the *effete* gene, which encodes the UBE2D orthologue Ubcd, affect wing formation (Pan et al., 2017). The specific roles of ubiquitination in Hedgehog signalling are less clearly defined in vertebrates, but the results of shRNA knockdown of UBE2D enzymes in vertebrate cells (Pan et al., 2017) together with the phenotypes observed for mutations in the *Drosophila* UBE2D orthologue Ubcd1 (Pan et al., 2017) all point to a significant role for UBE2D family members in vertebrate Shh signalling.

### Regulation of TGFβ signaling

The E3 ligase Arkadia (RNF111) acts as a positive regulator of TGFβ signaling in early mouse development by ubiquitinating the transcriptional repressor SnoN using members of the UBE2D family as E2 partners (Figure 4C) (Episkopou et al., 2001; Niederlander et al., 2001). Ubiquitination of SnoN targets it for degradation. SnoN inhibits expression of TGFβ target genes by interacting with Smad4 at gene promoters. Proteasomal degradation of ubiquitinated SnoN blocks this repression and allow phosphorylated Smad3 to interact with Smad4 and activate target gene expression (Levy et al., 2007). Results from knockout mice and ectopic expression in *Xenopus* embryos have shown that Arkadia-mediated regulation of TGFβ is involved in regulating dorsal/ventral specification, mesendoderm formation and head development in vertebrates. (Episkopou et al., 2001; Niederlander et al., 2001).

### Involvement of UBE2D3 in the NF-κB/IκB pathway

The canonical NF-κB pathway is regulated by the cytoplasmic inhibitor IκB, which sequesters NF-κB and keeps it in an inactive state (Henkel et al., 1993). Activation of NF-κB is triggered by phosphorylation-dependent ubiquitination of IκB, which results in degradation of IκB by the proteasome (Alkalay et al., 1995; Chen et al., 1995). Ubiquitination of IκB is mediated by the E3 ligase SCF^βTrCP^ in a reaction that uses UBE2D3 as the ubiquitin donor (Figure 4D) (Yaron et al., 1998; Gonen et al., 1999).

The zinc-finger protein A20 (also known as TNFAIP3) is a negative regulator of NF-κB, which acts to attenuate NF-κB-mediated inflammatory responses to tumour necrosis factor (TNF) and agents that stimulate Toll-like receptors (Lee et al., 2000; Boone et al., 2004). Part of the inhibitory effect is the result of interaction between A20 and conjugated UBE2D3, which has been reported to trigger ubiquitination and proteolysis of UBE2D3, blocking degradation of IκB (Shembade et al., 2010).

### DNA methylation

The E3 ligase UHRF1 has been shown to play an important role in promoting maintenance DNA methylation in mammals (Nishiyama et al., 2013; Qin et al., 2015). Recent work has shown that the UBE2D enzymes have an unusual mode of interaction with the ubiquitin-like (UBL) domain of UHRF1 and that this interaction is required for UHRF1-mediated ubiquitination of histone H3 (DaRosa et al., 2018). UHRF1 binds to di- and tri-methylated histone H3K9K9 (H3K9me2/3) on hemimethylated DNA and monoubiquitinates H3 at K14, K18, K23 and K27 (Figure 4E) (Harrison et al., 2016). The ubiquitinated residues act as recognition signals promoting the binding of DNMT1 to hemimethylated DNA and methylation of the unmethylated DNA strand (Nishiyama et al., 2013; Qin et al., 2015; Harrison et al., 2016).

### DNA repair

A screen of E2 enzymes using RNAi knockdown identified a number of E2s that affect DNA repair by end resection or homologous recombination (Schmidt et al., 2015). The UBE2D enzymes were among the E2s whose knockdown negatively affected DNA repair. The study identified an axis between the UBE2D enzymes and the E3 ligase RNF138, which leads to ubiquitination of C-terminal binding protein (CtBP) interacting protein (CtIP) (Figure 4F). CtIP co-ordinates homologous recombination- (HR) mediated DNA repair as part of a protein complex (CtIP/MRN) that initiates DNA end resection at double-strand breaks (You and Bailis, 2010). CtIP ubiquitination has positive and negative effects on DNA-resection. There is evidence that ubiquitination of the N-terminal region of the protein promotes recruitment of CtIP to DSBs (Schmidt et al., 2015) whereas ubiquitination of two lysines in the C-terminal region has been shown to inhibit phosphorylation of CtIP by the ATM kinase, which promotes DSB repair (Gao et al., 2020). These results highlight the complex roles played by ubiquitination in DNA repair. The results obtained by Schmidt et al., showing that several other E2s in addition to the UBE2Ds have non-redundant roles in promoting ubiquitination, emphasise the complexity of the roles played by ubiquitination in regulating DSB repair.

### Histone modification

Monobiquitination of core histones is a key regulatory modification that can signal activation or repression of gene transcription. Ubiquitination of lysine-119 of histone H2A (H2AK119) by the E3 ligases Ring1A and Ring1B in conjunction with the UBE2D enzymes is associated with transcriptional repression (Figure 4G). Ring1A/B are components of Polycomb Repressive Complex-1 (PRC1) which has essential roles in transcriptional regulation during early development and in a variety of different adult tissues (reviewed by Kuroda et al., 2020). The UBE2D enzymes have been shown to act as *in vitro* and *in vivo* ubiquitin donors for Ring1B-mediated ubiquitination (Bentley et al., 2011).

### Regulation of the cell cycle and apoptosis by p53

The p53 tumour suppressor protein functions primarily as a transcription factor that is involved in regulating a number of different stress responses in the cell. Rapid activation of p53 in response to a variety of stress stimuli, can result in several different effects including cell cycle arrest, apoptosis or senescence (reviewed by Chen, 2016). Post-translational regulation of the level of p53 by ubiquitination and proteasomal degradation of the protein plays an essential role in controlling p53-mediated responses (reviewed by (Hock and Vousden, 2014). Ubiquitination of p53 is mediated by the MDM2/MDM4 E3 ligase complex and makes use of UBE2D2 and UBE2D3 as ubiquitin donors (Figure 4H) (Saville et al., 2004; Yang et al., 2021). Separate knockouts of MDM2 and MDM4 give rise to early embryonic lethal phenotypes in mice, which can be rescued by knockout of the p53 gene (Jones et al., 1995; Parant et al., 2001). Within the MDM2/MDM4 complex, MDM2 functions as the primary E3 ligase in the p53 ubiquitination reaction. MDM4 (also known as MDMX) interacts with UBE2D2/3, recruiting them for use as ubiquitin donors for ubiquitination of p53 by MDM2 (Yang et al., 2021).

### Evidence that post-translational modifications are involved in regulation of the UBE2D proteins

**FIGURE 5:**
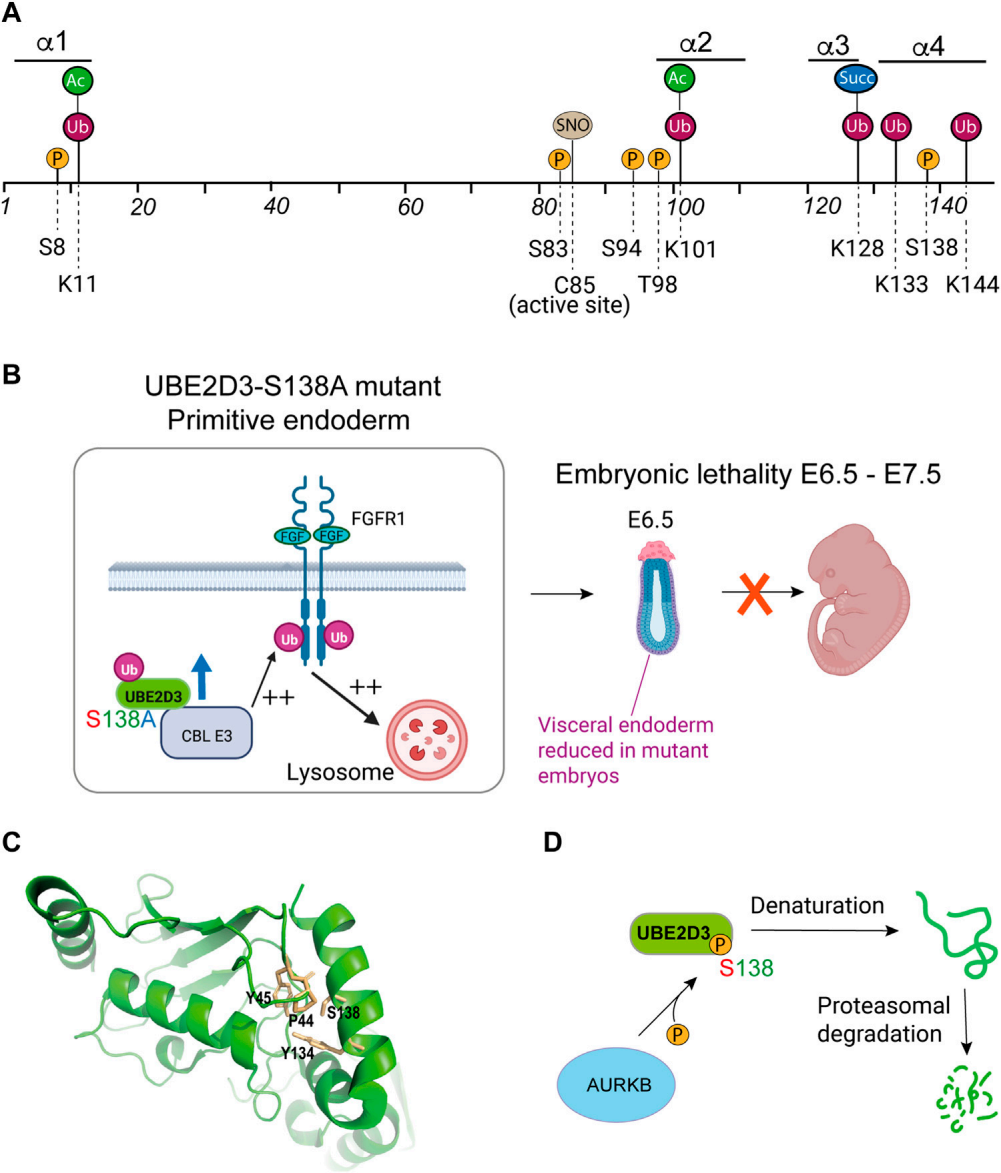
Role of post-translational modifications (PTMs) in the regulation of UBE2D enzymes. **(A)** Residues in mammalian UBE2D that have been shown to be targets for PTMs. P = phosphorylation; Ub = ubiquitination; Ac = acetylation; Succ = succinylation; SNO = S-nitrosylation. Numbers indicate the positions of amino acid residues. Information on PTMs was obtained from PhosphoSite^®^(Hornbeck et al., 2015), with the exception of S-nitrosylation of Cys85 (C85) (Valek et al., 2019; Fujikawa et al., 2020) and phosphorylation of UBE2D3-Ser138 (Roman-Trufero et al., 2020). **(B)** Phenotypic effects of the S138A mutation during early mouse development. Substitution of the amniote-specific Ser138 residue with the alanine residue that is found at this position in non-amniote species resulted in a 3-fold increase in the level of UBE2D3. In developing primitive endoderm (PrE), this results in increased ubiquitination of fibroblast growth factor receptor 1 (FGFR-1) by the CBL E3 ligase, which uses UBE2D3 as a ubiquitin donor. Enhancement of FGFR1 ubiqitination causes increased trafficking of the receptor to the lysosome leading to downregulation of FGFR1 signalling. This results in reduced levels of PrE and visceral endoderm and a high penetrance embryonic lethal phenotype between E6.5 and E12.5**. (C)** Proximity of residues within the hydrophobic core of UBE2D3 to S138. Phosphorylation of S138 would be predicted to disrupt the interaction with these residues and destabilise the protein. Image was prepared by visualising PDB: 3UGB using PyMol (v2.5.4). **(D)** Schematic representation of the effect of phosphorylation of the amniote-specific Ser138 residue by the Aurora B kinase on the stability of UBE2D3. Panels B and D created with BioRender.com.

E2 enzymes are subject to a number of different types of post-translational modification (PTM), including, ubiquitination, acetylation, S-nitrosylation and phosphorylation. Figure 5A shows that the PTMs that have been identified on mammalian UBE2D enzymes are grouped into two main clusters. One of these clusters overlaps the active site cysteine and part of the E3 binding domain, including the SPA motif (Ser94-Pro95-Ala96), which is present in a number of E2s. The SPA motif forms part of the E3 binding domain (Figures 2A, B) and has been shown to be involved in binding of UBE2D1 to the CHIP/STUB1 E3 ligase, which has essential roles in protein quality control through interaction with the chaperone proteins, HSP70 and HSP90 (Soss et al., 2011). The SPA motif has also been shown to be important for binding of UBE2D3 to the Polycomb protein RING1B (Bentley et al., 2011). Based on the analysis in these two studies, phosphorylation of Ser94 might be expected to affect the interaction of the UBE2D enzymes with CHIP, RING1B and related E3s, although whether binding is enhanced or reduced has not been tested.

Another PTM that forms part of this cluster is S-nitrosylation of the active site cysteine, which has been has been reported to reduce the activity of the UBE2D enzymes, with downstream effects on CHIP/STUB1 and on the targeting of misfolded proteins by endoplasmic reticulum-associated protein degradation (ERAD) (Valek et al., 2019; Fujikawa et al., 2020). S-nitrosylation has been implicated in pro- and anti-inflammatory effects, promotion of apoptosis and a wide range of other biological processes (Foster et al., 2003; Hara et al., 2005; Aguilar et al., 2020).

## Phosphorylation of UBE2D3-S138 regulates the stability and level of the protein

The second group of UBE2D PTMs is located in the C-terminal HTH region (Figure 5A) and includes a phosphorylation site at UBE2D3-Ser138 that is specific to UBE2D3 (Figure 2A) and is restricted to amniotes. Position-138 in UBE2D3 is unusual in that it is occupied by a highly conserved alanine residue across non-amniote eukaryotic orders including amphibia and fish, but is replaced by a serine that is fully conserved in the 78 amniote species that have been examined (Roman-Trufero et al., 2020). The substitution of serine at position 138, which appears to have occurred in the common ancestor to modern amniotes, generated a consensus site for the cell cycle kinase Aurora B. We have shown that Ser138 is phosphorylated by Aurora B in an *in vitro* kinase assay and also *in vivo* in mouse embryonic stem (mES) cells (Roman-Trufero et al., 2020).

We used genetic analysis in mice to investigate the functions of UBE2D3-Ser138 phosphorylation,. Ser138 was mutated to alanine in mouse ES cells and in mice using CRISPR/Cas9. Because alanine is the residue that is present at position 138 in amphibia and fish, this was effectively an experiment in reverse evolution. Our results (summarised in Figure 5B) showed that the introduction of the Ser138Ala (S138A) substitution in mice resulted in high penetrance embryonic lethality with death occurring at E6.5—E7.5, shortly after implantation. The mutant embryos had reduced amounts of visceral endoderm at E6.5. The effect of the mutation on extraembryonic endoderm differentiation was confirmed by showing that UBE2D3-Ser138Ala mutant mES cells showed greatly reduced differentiation into primitive endoderm (PrE) in an *in vitro* differentiation assay (Roman-Trufero et al., 2020). Evolution of the first amniotes is thought to have occurred during the Carboniferous period between 340 and 314 million years ago (Mya) (Carroll, 1964) (reviewed by Clack, 2012). Development of the extraembryonic tissues that form the membranes of the amniote egg was a key event in amniote evolution, PrE is one of the most ancient of these tissues and gives rise to the parietal endoderm on the inner surface of the yolk sac (reviewed by (Stern and Downs, 2012). The extraembryonic membranes of the yolk sac are critical for nutrient exchange in amniote embryos (Ferner and Mess, 2011; Sheng and Foley, 2012). PrE also gives rise to visceral endoderm, which has an important inductive role in amniote gastrulation (Stern and Downs, 2012). Our results suggest that the substitution of serine for alanine at position 138 in the UBE2D3 protein in the common ancestor of amniotes had a role in the evolution of amniote PrE.

Measurement of the level of UBE2D3 protein in the mutant S138A ES cells showed a 3-fold increase in the level of the mutant UBE2D3 compared with wild-type cells and with revertant ES cells generated by reverse A138S mutation of the mutant ES cells. These results implied that Ser138 phosphorylation destabilises UBE2D3 *in vivo*. This conclusion was further supported by the finding that phosphorylation of Ser138 by Aurora B in an *in vitro* reaction caused UBE2D3 to become insoluble and precipitate. Phosphomimetic mutant UBE2D3-Ser138Glu and Ser138Asp proteins were also found to be highly unstable compared with the wild-type protein when they were transiently expressed in ES cells and when they were expressed in bacteria. To better understand the mechanism behind this instability, *in silico* molecular dynamic simulation was used to show that Ser138 phosphorylation disrupts contacts involving Ser138 and hydrophobic residues in the interior of the UBE2D3 protein (Pro44, Tyr45 and Tyr134, see Figure 5C) and would be expected to cause denaturation and proteasomal degradation of the protein, (Figure 5D) (Roman-Trufero et al., 2020).

To examine the effects of the UBE2D3-Ser138Ala mutation on E3 ligases that use UBE2D3 as a ubiquitin donor, we used the proximity ligation assay (PLA) to measure the levels of interaction of UBE2D3 with the E3 ligase CBL after 3 days differentiation of PrE cells from wild-type and mutant mES cells. CBL catalyses ubiquitination and downregulation of the RTKs, PDFGRα and FGFR1, which are known to have essential roles in PrE differentiation. The results from the PLA showed that interaction between UBE2D3 and CBL is strongly increased in the Ser138Ala mutant cells, which have higher levels of UBE2D3 and that this effect is reversed in cells differentiated from the revertant ES cell line. The UBE2D3-Ser138Ala mutation also resulted in increased interaction between CBL and PDGFRα and FGFR1. Levels of both receptors were reduced in the mutant cells and the effect was reversed in the revertant cells (Figure 5B).

To further analyse the effect of the UBE2D3-Ser138Ala mutation on E3 ligase activity, we examined the level of H2AK119 ubiquitination which is mediated by the Polycomb protein RING1B and is associated with transcriptional repression. Ubiquitination by RING1B has been shown to use UBE2D3 as a ubiquitin donor (Figure 4) (Bentley et al., 2011). The results showed that the Ser138Ala mutant mES cells had increased levels of H2AK119 ubiquitination at a number of repressed and active genes, with a particularly strong effect observed at the promoters of the *Sox7* and *Gata6* genes, which encode proteins that are known to be master regulators of PrE differentiation. The effect was reversed in the Ala138Ser revertant cells.

Overall, these results lead us to conclude that the level of UBE2D3 protein is regulated by phosphorylation of UBE2D3-Ser138 during the early stages of mammalian development and that this regulation has an important role in PrE differentiation. They also indicate that the Aurora B kinase has a major role in generating this effect. Aurora B was originally identified as a cell cycle kinase which phosphorylates multiple substrates during mitosis (reviewed by (Carmena et al., 2012)) and is required for chromosome condensation and segregation, and for cytokinesis. Regulation of the cell cycle plays a key role in maintaining ES cell pluripotency and self-renewal, and differentiation of pluripotent cells is accompanied by changes to the timing and duration of cell cycle stages (reviewed by Padgett and Santos, 2020). There is also evidence that cell cycle kinases can be recruited for non-cell cycle-related functions that regulate pluripotency and differentiation. CDK1 has been shown to be involved in maintaining epigenetic marks genome-wide in ES cells (Michowski et al., 2020) and the Aurora A kinase has been found to regulate ES cell pluripotency and differentiation through phosphorylation of p53 and suppression of its role in ectodermal and mesodermal differentiation (Lee et al., 2012). The results described above indicate that Aurora B belongs to the category of cell cycle kinases that affect early embryonic development, with the added twist that the regulation of UBE2D3 by Aurora B first arose in the common ancestor to modern amniotes and could, therefore, have played a role in the evolution of the amniote embryo.

In addition to Ser138 phosphorylation, ubiquitination has been detected at three residues (Lys128, Lys133 and Lys144) in the UBE2D C-terminal region (Figure 5A). Ubiquitination of Lys128 is particularly interesting because this residue, which is highly conserved across eukaryotic species, has been implicated in recognition of lysine residues in substrates of the chaperone associated E3, CHIP/STUB1 (Kanack et al., 2020), and in ubiquitination of Lys119 of histone H2A by the Polycomb protein RING1B (McGinty et al., 2014). Succinylation, wubiquitination hich is involved in metabolic signalling (Mills and O'Neill, 2014), has also been detected at UBE2D3-Lys128 and would be expected to antagonise ubiquitination of this residue. The functional significance of the modifications at these positions has not been determined.

## Sequence conservation of the UBE2D enzymes across eukaryotic orders

**FIGURE 6:**
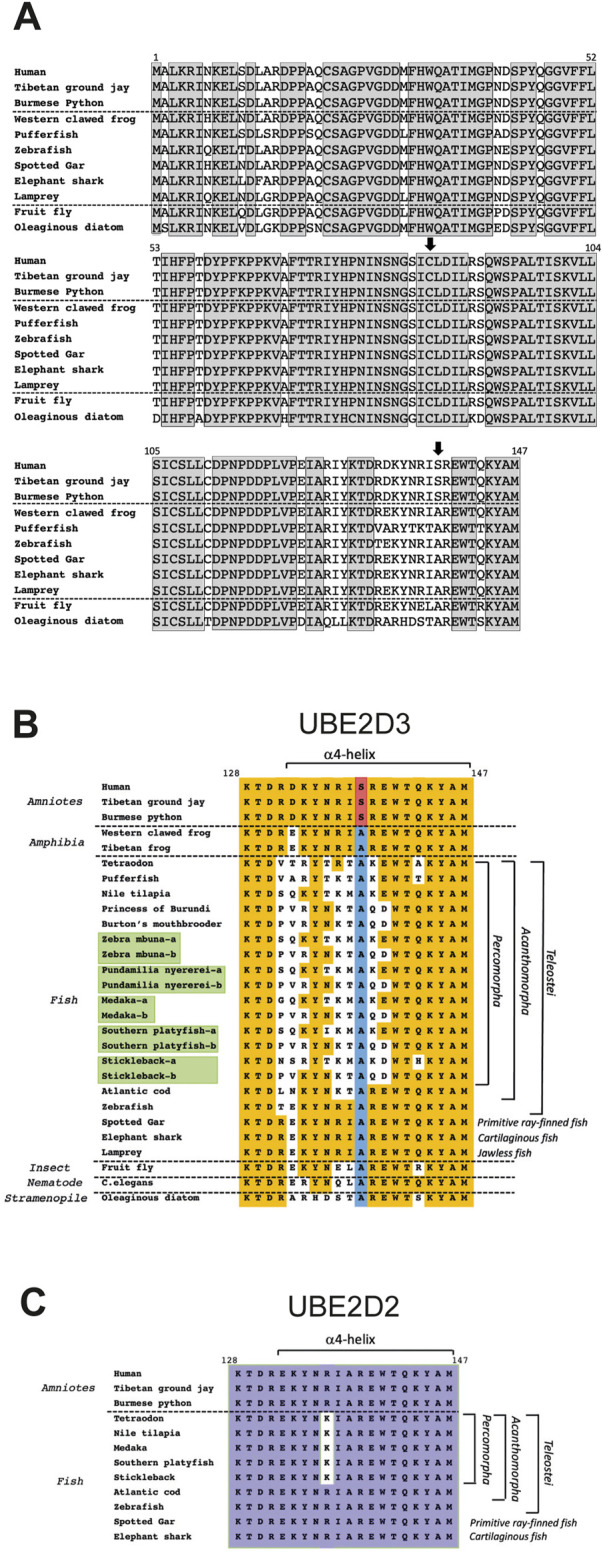
Comparison of the amino acid sequences of UBE2D3 orthologues across multiple eukaryotic lineages. **(A)** Comparison of the complete human UBE2D3 sequence with vertebrate and non-vertebrate orthologues. Grey shaded boxes indicate residues that are completely conserved across all of the species shown. Vertical arrows indicate the conserved active site cysteine (Cys85) and the amniote-specific phosphorylation target, Ser138. A full sequence comparison of UBE2D3 orthologues across 118 eukaryotic species can be found in Roman-Trufero et al. (2020). **(B)** Comparison of the amino acid sequence of the C-terminal region (residues 128–147) of UBE2D3 shows that part of the region is hypervariable in Acanthomorph fishes. Orange shading indicates sequence identity with the equivalent residues in the conserved amniote sequence. The Ala138 residue, which is conserved in all non-amniote lineages examined, is highlighted in blue. The serine residue that is found at this position in amniotes is highlighted in red. It is notable that the C-terminal sequences of jawless fish (lamprey), cartilaginous fish (elephant shark) and primitive ray-finned fish (spotted gar) show very high homology with tetrapod amphibian sequences (100%) and amniote sequences (90%) compared with the Acanthomorph sequences (55%–80%). The lowest homology with the ancestral jawless fish sequence (Lamprey) is observed in the Percomorpha (55%–70%). Duplicated UBE2D3 genes arising from the ancestral teleost genome duplication are highlighted in green and may have contributed to the hypervariability of these genes in Percomorph lineages by allowing subfunctionalisation or neofunctionalization of the duplicated genes. Comparison of the C-terminal sequences from the fruit fly *Drosophila melanogaster* and the nematode *C. elegans* also shows that these sequences have greater homology with jawless fish, amphibia and amniote UBE2D3 proteins compared with Percomorph fish. **(C)** Comparison of UBE2D2 C-terminal sequences provides evidence that UBE2D2 and UBE2D3 are under different selection pressures in Percomorph fish. Purple shading indicates residues that are conserved across the species examined. A number of fish species and all three frog species examined did not show evidence of a UBE2D2 gene (Roman-Trufero et al., 2020), suggesting that UBE2D2 may have been lost from these species.

An enigmatic feature of UBC-only E2 enzymes, including UBE2D family members, is the very high level of conservation of the protein sequences across multiple widely separated eukaryotic orders. Conservation of UBE2D3 was studied in detail by carrying out a protein sequence comparison across 118 eukaryotic species (Roman-Trufero et al., 2020). Examples from this comparison are shown in Figure 6A. The comparison showed that the amino acid sequence of UBE2D3 is 100% conserved across 78 amniote species. The conserved amniote sequence showed 97% homology with sequences from frogs and cartilaginous and primitive ray-finned fish, 94% homology with the Ubcd1 gene from fruit fly *Drosophila melanogaster* and 81% homology with the orthologous sequence from *Fistulifera solaris*, a single celled oleaginous diatom species that belongs to the Stramenopile group. The estimated time of divergence of the Stramenopiles from the lineage that led to metazoans is between 1.0 and 1.5 billion years ago (Brown and Sorhannus, 2010), highlighting the extraordinary conservation of the UBE2D proteins during eukaryotic evolution.

In addition to the role of the amniote-specific Ser138 residue in regulating the level of UBE2D3-dependent ubiquitination during development, evidence of a role for UBE2D3 in vertebrate evolution has come from the observation in Acanthomorph fishes of an exception to the high conservation of UBE2D3 across eukaryotic orders (see Figure 6B and accompanying legend). The Acanthomorpha are a highly successful group of teleost fishes that comprise one-third of all vertebrate species and are notable for their morphological diversity (Helfman et al., 2009; Chen et al., 2014). Much of this diversity stems from the explosive radiation of an Acanthomorph subgroup, the Percomorpha, that took place in the wake of the Cretaceous/Paleogene mass extinction, which occurred 66 Mya (Friedman, 2010). The Percomorpha include pufferfish, seahorses, sticklebacks and the rapidly evolving cichlid species flocks that are endemic to several large east African lakes (Helfman et al., 2009). Percomorph species examined show an exceptional level of hypervariability in the C-terminal region of UBE2D3 relative to other vertebrate species (Figure 6B), raising the interesting possibility that changes to UBE2D3 regulation and the signalling pathways that lie downstream from it could have contributed to the diversity of this order. It is notable that the variation in the C-terminal region is restricted to UBE2D3. Comparison of available UBE2D2 sequences showed only a single conservative (Arg to Lys) substitution (95% identity) in a comparison of the C-terminal region in Percomorph species with basal fish species (Figure 6C). This contrasts with the 55%–75% identity observed in the equivalent comparison of UBE2D3 sequences (Figure 6B). It is noteworthy that the UBE2D3 residues that are hypervariable in Percomorph fishes are located predominantly on the outward-facing surface of the α4-helix and the turn region of the HTH motif, where they would be available to form novel protein-protein contacts. Such contacts could potentially affect the activity of UBE2D3 (Welsh et al., 2022).

UBE2D3-dependent ubiquitination of the Gli2/3 transcription factors is known to antagonise Hedgehog signalling (see Figure 4 and accompanying legend). Artificially modifying the level of Hedgehog signalling in the jaws of Zebrafish has been shown to alter craniofacial bone plasticity in response to environmental challenges (Navon et al., 2020). This provides a potential mechanism by which modulation of UBE2D3 activity might have facilitated changes to jaw structures during Percomorph evolution. Cichlids undergo rapid evolution of different feeding strategies through the acquisition of diverse craniofacial structures (Matthews and Albertson, 2017) and it is notable that cichlid species show some of the highest variability in the C-terminal region of UBE2D3 among the fish species that have been examined (Figure 6B). These ideas are obviously highly speculative, but they are potentially testable.

## Involvement of the UBE2D enzymes in human disease

The UBE2D enzymes act as ubiquitin donors for a number of E3 ligases that are involved in human disease. These include the RBR E3 ligase Parkin, which is a major source of recessive mutations that give rise to Parkinson’s disease (Fiesel et al., 2014) and the RING E3 ligase CHIP/STUB1, which is mutated in several subcategories of hereditary spinocerebellar ataxia (Heimdal et al., 2014; Kanack et al., 2018). UBE2D enzymes are also implicated in inflammatory pathways by virtue of their role in activating NFκB through downregulation of IκB. A small molecule inhibitor of UBE2D activity has been reported to inhibit inflammatory activity in a mouse model for inflammatory disease (Liu et al., 2014).

UBE2D enzymes are critical in several cellular pathways involved in cancer progression, including NF-κB, TGFβ and p53 (discussed in this review) and there is abundant evidence in the literature showing dysregulation of UBE2D enzymes in multiple cancer types (reviewed by Zhou et al., 2020). Moreover, when consulting the Cancer Dependency Map (DepMap, https://depmap.org/portal/), we found that over 80% of the cell lines (879 out of 1,086 cell lines analysed) in the database are dependent on UBE2D3 (DepMap 22Q2 Public + Score Chronos). UBE2D enzymes would also be expected to have tumour suppressor activity *via* their role in downregulating RTK function *via* the CBL E3 ligase. Therefore, understanding the fine regulation of UBE2D enzymes, and other E2-conjugating enzymes, should provide new routes towards the development of effective anti-cancer therapeutic strategies.

## Conclusion and future perspectives

The importance of the UBE2D enzymes for the functioning of eukaryotic cells is highlighted by their involvement in a wide-range of processes including major signalling pathways and the regulation of protein turnover in the cell. The ability of the UBE2D family to interact with a large number of E3s has sometimes led to the perception that their lack of specificity precludes them from having regulatory roles. However, there are many examples of proteins that are involved in a broad range of biological processes but are able to confer regulatory specificity through the action of PTMs, cellular compartmentalisation, variation in level, and interactions with other proteins. Our results showing that phosphorylation can destabilise UBE2D3 suggest that the levels of the UBE2D proteins in the cell are critical for correct regulation of their function and that PTMs that affect their stability can directly affect their downstream functions. These findings further support the idea that control of the stability and level of UBE2D3 is finely tuned, providing a means for regulating ubiquitination by E3 ligases that use it as a ubiquitin donor.

The importance of regulating levels of E2 enzymes has interesting implications when considered in conjunction with the observation that many E3 ligase enzymes make use of several E2 enzymes, which show different degrees of redundancy. The robustness provided by redundancy of some E2 enzymes is not surprising, given that they affect the activity of multiple E3s but it is also means that varying E2 levels can provide subtle levels of regulation of ubiquitination. This is illustrated by our observation that the increase in the level of UBE2D3 when phosphorylation is blocked, results in increased downregulation of RTKs by CBL (Roman-Trufero et al., 2020) despite the fact that it has been shown that CBL uses other E2s in addition to UBE2D3 (Liyasova et al., 2019). Understanding the complex functional and regulatory effects of multiple E2 usage and the effects of PTMs on this regulation will be one of the challenges for future research in this area.
